# Understanding the sustainability of implementing HIV services in criminal justice settings

**DOI:** 10.1186/s40352-015-0018-2

**Published:** 2015-03-07

**Authors:** Christy A Visher, Yang Yang, Shannon G Mitchell, Yvonne Patterson, Holly Swan, Jennifer Pankow

**Affiliations:** 1 Center for Drug & Health Studies, Department of Sociology and Criminal Justice, University of Delaware, Newark, USA; 2 Department of Psychology, University of Louisiana at Lafayette, Lafayette, USA; 3 Friends Research Institute, Baltimore, MD USA; 4 University of Connecticut, Storrs, USA; 5 Center for Healthcare Organization and Implementation Research (CHOIR), Edith Nourse Rogers Memorial VA Medical Center, Bedford, USA; 6 Institute of Behavioral Research, Texas Christian University, Fort Worth, USA

**Keywords:** Implementation, Sustainability, HIV services, Correctional facility, Change team

## Abstract

**Background:**

In the growing field of implementation science, sustainability is a critical component of the implementation process of moving evidence-based treatments to regular practice. This paper is intended to extend our understanding of factors that influence the sustainability of HIV services in correctional settings following an organization-level intervention designed to implement improvements in preventing, detecting, or treating HIV for persons under correctional supervision.

**Methods:**

Using semi-structured interviews to elicit perceptions from the principal researcher and executive sponsor at each of nine participating sites, this study explores the variations in the sustainability of HIV services in these criminal justice settings following the experimental implementation intervention.

**Results:**

In six of the nine sites, changes in HIV services implemented as a result of the organizational intervention were sustained six to nine months following the end of project implementation. Organizational endorsement at multiple levels is likely the principal factor that facilitates sustainability.

**Conclusions:**

The factors that result in the sustainability of changes to health services in correctional organizations include elements internal and external to the organization. Implementation strategies, such as the change team model strategy used in this study, are also sustainable and can be used to identify other changes that could be made, or improve other aspects of service delivery.

Evidence-based public health programs can deliver positive health outcomes, but only if they are able to sustain these actions over time. Although there is growing evidence about the types of interventions that may be successfully implemented (Grimshaw et al. 2012; Michie et al. 2009), there is considerably less knowledge regarding how and why new ways of working are sustained and become established in everyday practice (Aarons et al. 2011). This question of sustainability is critical to translating research on evidence-based practices into routine operations.

## Background

In the implementation and dissemination literature, sustainability is considered a stage in an overall cycle of intervention development, adoption, implementation and adaptation (Proctor et al. 2009, 2011); thus, it is useful to consider sustainability in relation to the earlier stages. Proctor et al. (2009) regard sustainability as a type of implementation outcome as distinct from service or client outcomes (e.g., satisfaction, symptomology) that results from the process of using evidence-based implementation strategies to establish new evidence-based treatments or services. The dynamics involved in the adoption and implementation stages often extend to the stage of sustainability.

The existing literature on organizational change and the diffusion of innovations emphasizes that all of these processes are often affected by the complex nature of the organizational environment including external, institutional, and political forces (Fitzgerald et al. 2002; Dopson et al. 2002). Factors influencing sustainability include (1) characteristics of the intervention, particularly its flexibility, cost, and effectiveness; (2) factors in the organizational setting, such as whether the intervention is a good fit with the organization, presence of an internal champion to advocate for the program, organization capacity and leadership, and attitudes of key staff; and (3) factors in the community environment, including partnerships and continued funding. Scheirer and Dearing (2011) point out that sustainable outcomes for public health programs are likely to require processes that start long before the end of initial implementation and program funding.

### Operationalizing sustainability

Definitions and the measurement of sustainability have also received increasing attention in the implementation and dissemination literature. According to Schell and colleagues, sustainability is not simply the result of a process but is action-oriented: the “ability to maintain programming and its benefits over time” (2013:15) and includes the set of organizational and contextual factors that build capacity for maintaining a program. These scholars identified nine domains of sustainability from a comprehensive literature review: political support, funding stability, partnerships, organizational capacity, program evaluation, program adaptation, communications, public health impacts, and strategic planning (Schell et al. 2013). These domains further divide into factors internal to the program and factors external to the program. However, in a different review of 125 papers on sustainability, only 35 percent included a formal definition, which focused on the continuation of programs and practices in organizations, systems, and communities during a post-implementation period (Wiltsey Stirman et al. 2012).

Other researchers want attention paid to specifying the achievements that have been made in sustaining programs. Scheirer and Dearing (2011) argue that research on sustainability needs to move beyond a simple dichotomy of measuring: “Did the program continue or not? (p. 2061)”. Other types of implementation outcomes might include continuation of benefits or outcomes for clients; continuation of program activities of original intervention; maintaining community-level partnerships developed during the intervention; maintaining new organizational practices, procedures, or policies; sustaining attention to issue or problem; and program diffusion and replication in other sites. There does not appear to be any consensus on which type of sustainability outcome should receive priority for research. Thus, a clear need exists for more research on sustainability outcomes and the factors that influence these outcomes following implementation of evidence–based interventions (Proctor et al. 2011; Wiltsey Stirman et al. 2012).

This paper reports the results of a sustainability substudy that was conducted as part of a larger implementation project. The larger project was conducted in order to gain a better understanding of the processes that promote the integration of evidence-based, substance abuse treatment and HIV services into criminal justice settings. Individuals incarcerated in U.S. prisons and jails are at high risk for mental health disorders, substance abuse disorders, and physical health problems such as HIV/AIDS, hepatitis, and other chronic and infectious diseases (Maruschak 2012). For individuals with HIV/AIDS, early detection and linkage to treatment are essential best practices for managing the disease and reducing the risk of transmission (Centers for Disease Control and Prevention 2003). Unfortunately, HIV services in prisons and jails suffer from lack of adherence to best practices in HIV testing, prevention, and treatment access (Beckwith et al. 2010; Booker et al. 2013; Springer and Altice 2005). Thus, it is important for correctional facilities to close these gaps in service delivery by initiating and maintaining evidence-based programs that improve access to testing for HIV, enhance prevention through education programs, and facilitate linkages to community-based HIV services for individuals exiting confinement.

In 2008, the National Institute on Drug Abuse (NIDA) initiated the second phase of the Criminal Justice Drug Abuse Treatment Studies (CJ-DATS 2) with a multisite cooperative of research centers across the U.S. CJ-DATS 2 protocols targeted three areas of service delivery to persons under correctional supervision (in prison or jail) with substance abuse problems: (1) assessment and case planning; (2) medication-assisted treatment options; and (3) HIV services (see Ducharme et al. 2013). This paper is concerned with the third protocol of the CJ-DATS project, HIV Services and Treatment Implementation in Corrections (HIV-STIC), and the sustainability of the outcomes from that study.

## Methods

To gain a better understanding of the processes that promote implementation of evidence-based HIV services in correctional settings, the HIV Services and Treatment Implementation in Corrections (HIV-STIC) project utilized a multisite, cluster randomized trial to test an organizational process improvement strategy (Belenko et al. 2013). Each of nine research centers was paired with a criminal justice partner agency, generally a state- or county-level jail or prison agency. The experimental sites initiated improvements in evidence-based HIV services using a local change team (LCT) approach involving staff from correctional, medical, and community health agencies. With the help of a trained coach, the change teams were expected to engage in several actions, including a needs assessment, strategic planning, and a series of rapid cycle process improvement activities designed to improve either HIV testing, prevention, or linkage to care.

The HIV service improvements implemented as a part of the HIV-STIC protocol varied considerably across research centers (Belenko et al. 2013). The change teams selected a variety of HIV services for improvement during the course of the intervention. Some examples include increasing HIV prevention attendance among female inmates; increasing the percentage of inmates receiving an HIV test at admission; and improving the linkage to community treatment for HIV positive inmates leaving prison.

The majority of the literature on sustainability has investigated the implementation and sustainability of community programs promoting public health. Several factors deemed crucial in community programs may have a different impact on services implemented in a correctional setting, which is a highly organized environment with distinctive priorities (i.e., public safety). The primary aims of the sustainability and success substudy were to carry out qualitative, comparative case-study research at the nine research sites to better understand: (1) organizational systems change and sustainability of improvements in HIV services in correctional agencies, and (2) organizational characteristics and processes that lead to “successful” and “unsuccessful” implementation. This paper addresses the first aim and reports on the findings about the sustainability of the HIV services improvements using interviews with the principal researcher at each center and lead administrator of their partner criminal justice agency six to nine months after implementation ended. The perceptions of the key informants who design and deliver the implementation are important for understanding pertinent factors related to service sustainability.

### Research questions

The principal research question that this study sought to address was the following: What helps and hinders the medium-term sustainability of improvements in HIV services in correctional settings? Specifically, this study aims to explore three facets of sustainability: (1) What changes in the implementation process and outcome have been sustained? (2) To what extent has sustainability of policy or practice changes been achieved? (3) What factors were perceived by researchers and practitioners as facilitators or barriers that influenced the sustainability of implemented practices as well as the degree of sustainability?

### Participants

The research center principal investigator (PI) and the lead administrator, or executive sponsor (ES), (or their designees) in each of the nine study sites were recruited to participate in an in-depth, semi-structured telephone interview. In the HIV-STIC protocol, an ES is an agency-level administrator who determines the intervention focus in their state or county and passively monitors local progress without being involved in day-to-day management of the project implementation (Belenko et al. 2013). In the nine sites, these individuals held positions such as correctional medical director, chief medical executive, health care administrator, deputy commissioner of support services, and state infectious disease physician for corrections. PIs and ESs from nine research sites were contacted; nine PIs and eight ESs participated in the interviews (one ES was no longer with the organization by the time the interviews were conducted).

### Procedures and measures

The semi-structured interview focused on each respondent’s evaluation and perception of the implementation of the HIV-STIC intervention in their respective study sites. Examples of topics covered in the interview pertinent to the current study included: (a) the impact of the protocol on each study site, (b) elements that had been sustained and why, and (c) lasting changes to the delivery of HIV services. The semi-structured interview guide was approved by the Internal Review Board at each research center. Six to nine months following the completion of HIV-STIC implementation, PIs and ESs were recruited for an interview, which was conducted by a senior qualitative researcher via telephone. Each interview lasted about one hour. Informed consent was obtained prior to each interview. Interviews were transcribed verbatim and imported into Altas.ti for analysis.

### Analyses

Inductive coding was used to analyze the qualitative data, which generated seven primary codes: (1) change team issues, (2) communication, (3) environmental factors, (4) focus of intervention, (5) success, (6) sustainability, and (7) the appropriateness of the local change team process. Filtering on the primary code “sustainability,” data were further inductively coded, which produced three secondary codes: (1) tangible elements of sustainability, (2) peripheral elements of sustainability, and (3) factors associated with sustainability. Two primary coders, supervised by a senior qualitative researcher, were involved in the primary and secondary coding processes. During the primary coding stage, two coders first coded four transcripts (two PI transcripts and two ES transcripts) independently and then discussed the discrepancies and consolidated the codebook. Afterwards, coders split work but continued the discussion and debriefing during the entire coding process. The process was repeated during the secondary coding stage. In data analysis, three qualitative researchers (including the two primary coders) immersed themselves in the data and discussed the emergent themes until consensus was reached. The data analysis yielded three main themes and several subthemes, which are explicated in the following section.

## Results and discussion

Analysis revealed that during the months after implementation a majority (6 out of 9) of the research sites were able to sustain the practices or processes that were introduced through HIV-STIC, although the degree and type of sustainability varied. Three types of sustainability were reported by study participants: sustainability of changes to HIV services, sustainability of the local change team implementation strategy, and sustainability of other peripheral elements related to the HIV-STIC study. In addition to variation in the type of sustainability that was reported, study participants indicated that the degree of sustainability was also multifaceted in that new practices or processes were adopted at both the agency-level and the system-level of the site. Finally, participants reported several factors that influenced the sustainability of changed HIV services. Each of these themes is discussed.

### Sustaining changes to HIV services

Respondents from several sites stated that they were able to continue providing the services related to the focus area (i.e., prevention, testing, or linkage to treatment) that they implemented during the HIV-STIC study. Changes were sustained either by codifying the changes into system procedures or institutionalizing them into a system policy. As an example of codifying new practices into a system procedure, the PI from a site that focused on the linkage between prison-based agencies and community treatment providers stated that:

*The other thing that they did was to get meetings scheduled with the providers and have…attendance at those meetings become a part of their probation and parole guidelines so that they actually had to do that as part of their… supervision plan.*

In this example, a new process for enhancing the linkage process -- meeting with the community provider-- was codified into the standard procedures for the probation/parole officers to follow in developing their supervision plan.

At several sites, changes in the provision of HIV services were sustained because the implemented changes had been institutionalized into the system policy. For example, one PI from a site focusing on enhancing the linkage to treatment said

*The…community partner and the change team leader created a communications form to fax back and forth between the two agencies. And…all of that work got instituted into DOC policy.*

In addition, some sites were able to disseminate the changes into a broader system context and reach a greater scope. For example, the ES of a site focusing on the linkage to treatment said:

*Whatever the intervention team produced if it was good we used it in both facilities [i.e., a male facility and a female facility]… I mean the intervention was finished by the time we got around to understanding this aspect of the problem [in the process for scheduling appointments] and investigating it in detail… We actually went [live] with our [new] pilot electronic medical records two weeks ago. And that’s at all the facilities here that serve females, [in] three different facilities.*

For this site, changes to practices concerning linkage to HIV treatment (i.e., scheduling) were piloted as part of HIV-STIC. They started with implementing an intervention in one facility and expanded the intervention to the control group. In the few months after implementation, these changes were scaled-up into the broader system context across three different facilities. This scaling-up of the practice points to this site’s ability to sustain the improvements.

### Sustaining the local change team implementation strategy

In addition to sustaining the actual changes to HIV services, participants also highlighted the value of the Local Change Team (LCT) model to the sites and noted how its use was sustained beyond the HIV-STIC study as a strategy for continuing to improve HIV services. Additionally, in some sites, the LCT model was used for implementing changes to other service areas in the criminal justice agency. For example, a PI reported how their criminal justice partner applied the LCT model for promoting other health services:

*Our Executive Sponsor and his bureau [buy into this LCT model and] they’ve actually discussed using this model for other issues in their system. [Administrators in the] healthcare agency wanted to use the change team model to figure out linkages for other chronic health issues, not just HIV.*

The sustainability of the LCT model was particularly noteworthy when the LCT model was “scaling out” (Hendricks Brown 2014) in a correctional setting (e.g., into a new environment with a different mission). For example, an ES in one research site noted that:

*I’ve [been] promoted to another facility that’s much larger and [has] a much different mission [i.e., intake department] than where I was before. And so it’s a completely different focus; it’s a completely different mission and there are things that I have implemented in processes [in the new facility] and you know it’s [using] the change model and it’s served me well [in my new role].*

### Other peripheral elements related to the HIV-STIC study

During HIV-STIC, change team efforts were focused on improving HIV services. However, during this process, several peripheral elements emerged as a result of the organizational intervention, including the establishment of networks between different stakeholders, and enhanced collaboration and coordination between different agencies. These peripheral elements are implicitly associated with the way agencies provide treatment services, and sustaining these elements often led to improved quality of client services. One ES stated that:

[*After the implementation ended], they were [still] using [the relationships with the community providers]… and [keeping] in contact with [those community providers] from our medical field prison…when making referrals.*

Likewise, one PI at another site reported that the HIV-STIC project brought corrections and community providers together and the communication persisted after the implementation ended.

*Once the project was over that relationship [between corrections and community providers] still existed and our change team leader actually got promoted to the prisons and she took that relationship with her and is now training her staff at the prisons to work with that community provider and go visit the community facility and get to know those people.*

### Factors influencing sustainability of changes to HIV services

In addition to identifying several facets of sustainability as it related to the HIV-STIC study, analyses identified several factors deemed critical for successfully sustaining implemented changes as well as barriers that were associated with sustaining these changes. These factors can be categorized into three themes: organizational endorsement, resources, and champions.

### Organizational endorsement

Organizational endorsement, a theme composed of administrative and staff buy-in, emerged as a prominent facilitator to sustaining implemented changes to HIV services. Administrative buy-in refers to administrative-level acknowledgement of the value of the implemented changes and willingness to actively support the continuity of these changes. For example, one ES who confirmed that changes were sustained at his site stressed the value he places on service linkage:

*I keep saying it’s because it’s the continuity of care that we want to keep intact…And that gives us satisfaction and also makes us feel comfortable that when we are releasing our offender, making sure that [the offender] has full knowledge [of where he would receive his aftercare treatment].*

In this example, administrative buy-in facilitated the sustainability of the intervention because this ES understood the value of executing the service changes and was invested in sustaining them.

Another participant, a PI, cited the importance of staff buy-in during project implementation for promoting the sustainability of implemented changes:

*The executive sponsor just said “yep this is what we’re doing from here on out.” And the folks that we involved, and the nice part is that [ES] doesn’t even have to filter that down because the folks involved from the change team and even the person in the control group at the other institution are right there, they already know about it…The folks that are in charge of it were already involved and so they’re already on board and it just becomes the procedure of the institution moving forward because they’re already working on it themselves in a sense.*

Given the dynamic organizational context where the implementation took place, the organizational endorsement was not always granted and a lack of buy-in became a barrier to sustainability. For example, one PI identified the incompatibility between implemented changes and the organization’s strategic priorities as a barrier to systematic dissemination and sustainability:

*When you look at the system’s priority, not the correctional health services around HIV but the system’s priority, that’s not where they are right now because right now what they are seeing is that they are understaffed*.

At this site, insufficient organizational endorsement resulted from a mismatch between the project’s goals and the current organizational focus, which prevented the sustainability of changes to HIV services implemented during the HIV-STIC study. In addition, this quote illustrates the influence of organizational resources in the sustainability of implemented practices, a prominent theme that is explicated in the following section.

### Resources and staff continuity

Participants from several sites indicated the importance of continuous funding for sustaining services and the constraints of the lack of resources (including human resources) on sustaining the implemented changes. For example, one ES noted that changes were discontinued when the HIV-STIC study was over due to a lack of funding:

*It is on the shelf and I continue to bother the DOC [Department of Corrections] to see if they will fund it. They kept saying “yes,” “maybe,” “yes,” “maybe”. And then I’m still working with [another agency] with the hopes that they can write a grant. The problem is that I’m overworked enough I don’t have the time to write grants right now and so that’s where it kind of fell flat.*

In this example, job priorities also served as a prominent barrier wherein staff’s regular job responsibilities competed with the efforts in sustaining the changes. This alludes to another crucial factor in determining the sustainability of service changes: the availability and involvement of key players. In fact, several sites experienced administrative and line-staff turnover as a barrier to carrying on the implemented changes, as noted by one ES:

*The person who replaced me, I think she was brand new to the position, I think she had so much else on her plate, so to speak, with learning the new job and it’s hard to say.*

At this site, the ES’s job transition exerted a negative influence on the continuity of practice adoption, which is in sharp contrast to the facilitative effect of organizational endorsement from the administrators and staff who remained in their positions.

Likewise, the ES from another site was concerned about the staff turnover at the healthcare agency which may have produced the discontinuity of practices.

*So, even if you have the policy in place, even if you have the protocol in place, if you go through three people within the positions and contracted health care is like that, you have constant turnover, you know you’re going to continue to have [turnover], those issues.*

### Champion

Disseminating evidence-based practices in service organizations can range from “letting it happen” to “making it happen,” depending on the degree of involvement by stakeholders (Greenhalgh et al. 2004). Indeed, participants highlighted the influence of administrators and LCT leaders who, beyond just buying-in and supporting the implementation efforts, actually served as a champion for the sustainability of implemented changes. For example, one PI stated that the ES at their site advocated for sustaining and scaling-up the changes to more sites:

*The executive sponsor in our state was aware of all the changes that were made and, as many might be helpful in other settings, she moved to suggest them and to have those kinds of changes made elsewhere.*

Moreover, administrators and LCT leaders took advantage of their professional experience and knowledge of the structure and operation of healthcare agencies in correctional settings to proactively identify the factors, especially potential barriers, pertinent to service sustainability. Illustrating this point, one PI said:

*[The change team leader] kept making that very clear along the whole way, to me and to the team and to everybody she was saying because her whole thing was we have to get the stuff in the policy… That was her pushing that this needs to be DOC policy. This needs to be DOC policy, which doesn’t need to be this contractor’s practice. This needs to be DOC policy.*

Clearly, this LCT leader employed her professional vision and influences to strategically promote the continuity of changes that emerged from the HIV-STIC organizational intervention.

## Conclusions

Despite efforts being made to reduce the gap between evidence-based practices and routine practices (e.g., Proctor et al. 2009; Greenhalgh et al. 2004), there remains a lack of literature regarding successful sustainability and dissemination of organizational changes in health-related settings (e.g., Goisman et al. 1999; McHugh and Barlow 2010; Stewart and Chambless 2007). Limited research has identified the factors that influence sustainability including characteristics of the intervention, organizational factors, and contextual factors (e.g., Scheirer and Dearing 2011). More research is needed, particularly regarding the factors that influence the implementation of health services in a criminal justice setting. Anchored in the HIV-STIC protocol, this study shed light on what elements were sustained and factors that facilitated or hindered the sustainability of the intervention across nine research sites, using principal investigators and lead agency administrators as key informants.

Six out of nine research sites reported that the implemented services had been either codified at the site or institutionalized into agency (i.e., Department of Correction) policy at the site, or scaled up to a broader context in terms of extending the services or practices to other facilities. In addition, participants reported a peripheral element of sustainability—improvements in the relationship between different agencies providing HIV services. Inter-organizational collaboration and communication, which are opportunities for learning and information sharing in the pursuit of service enhancement, are believed to promote implementation processes and therefore quality of services provided in organizations. The relationships established during the course of implementation, either as a focus area of the intervention (e.g., linkage to treatment) or an inevitable component peripheral to implementation is a critical element of sustainability. Additionally, inter-organizational relationships developed during the HIV-STIC study strengthened the partnership between multiple organizations and facilitated the communication of organizational values and missions, which in turn led to alignment of practices and eventually better quality of care.

There is a dearth of research investigating the factors that influence sustainability of health practices. The current findings showed that the sustainability of health services is contingent on organizational endorsement (including administrative and staff buy-in), staff continuity, and having a champion driving the implemented services, whereas a mismatch between intervention and organizational priorities, a lack of resources, and staff turnover served as barriers to sustaining practices. Accordingly, the lack of sustainability in three sites in this study was linked to one or more of several factors: the incompatibility of the implemented services with organizational priorities, staff turnover (especially among those who had participated in the intervention), and lack of funding to sustain the implemented services.

Organizational endorsement at multiple levels is likely the principal factor that facilitates sustainability. Several sites identified that the buy-in and support from both administrators and staff were crucial to maintain the new or improved HIV services. When the value of practices was salient to administrators or congruent with the organizational mission, administrators tended to buy-in and put efforts in place to sustain the practices. The buy-in of the staff, particularly those who had been engaged in the implementation process, also facilitated sustainability of the intervention because they actually served as the front-line workforce who continued those practices during the post-implementation period.

The sustainability of implemented services depends on the congruence with organizational priorities. We found a lack of sustainability for sites where organizations set different priorities than goals of the implemented services. In particular, all three sites which did not continue implemented services did not view the services as a core component of the staff’s job responsibilities. Therefore, the compatibility of organizational priorities must be considered in order to ensure the applicability of implementation outcomes across a range of settings (Proctor et al. 2011).

Having an administrative-level champion who maneuvers in a dynamic, complex organizational structure and drives forward the sustainability of implemented practices is also important. In the sites where practices were sustained, the executive sponsor either directly requested the scaling up of practices to a broader system or strategically disseminated those practices (e.g., making the implemented practices part of existing agency policy). This is consistent with previous research indicating that having a champion with a managerial position who advocates for the intervention is necessary to maintain changes (Goodson et al. 2001; Greenhalgh et al. 2004; Wandersman et al. 2000).

One of the most commonly cited factors that hinder service sustainability is the availability of resources, particularly the project funding (Blasinsky et al. 2006; Wiig et al. 2010), which also emerged in the current study. A deficiency in funding and issues with staffing, particularly prominent in sites which discontinued the implemented services, resulted in a lack of sustainability for the implemented HIV services. With staff turnover and work overburden, there was a lack of human resources to effectively continue the implemented practices, which was in turn detrimental to service sustainability. Staff who had been trained during the implementation played a critical role in sustaining the implemented practices.

Another interesting, but rarely discussed outcome in the sustainability literature is the sustainability of implementation strategies. In addition to the endurance of actual implementation outcomes, several sites have reported that they have extended or planned to extend the LCT model to improve services designed for other health-related problems. This indicates that stakeholders have not only acknowledged the value of improved HIV services and practices in a correctional setting but also adopted the organizational approach for implementing the practices. This finding is consistent with Burnes and Jackson’s (2011) argument that the success of interventions relies on the acceptance of the content of the implementation as well as the approach used for implementation. The sustainability of the implementation strategy is particularly crucial when the availability of resources (e.g., funding, staff resources) does not allow for the continuation of implemented practices, but the implementation strategy or model still survives or penetrates through the system to identify other changes that could be made, or improve other aspects of service delivery.

### The significance of the HIV-STIC project

This paper’s analysis of policy and practice changes that were sustained as a result of the HIV-STIC change team intervention reinforces the study’s findings on the positive impacts of the intervention on both service delivery and staff attitudes (see Pearson et al. 2014; Visher et al. 2014). The change teams selected a variety of HIV services for improvement during the course of the intervention, including increasing HIV prevention attendance among female inmates; increasing the percentage of inmates receiving HIV education just prior to release; increasing the percentage of inmates receiving an HIV test at admission; increasing overall HIV testing; improving the linkage to community treatment for HIV positive inmates leaving prison; reducing wait times and no-shows to the community HIV treatment provider; improving continuity of anti-retroviral therapy medications for inmates leaving prison; and expanding peer-led HIV prevention programs (Belenko et al. 2013). Using a prospective meta-analytic design, analysis of these outcomes revealed an overall positive effect that was statistically significant, with intervention facilities delivering more HIV services (prevention, testing, and/or linkage to treatment) for offenders under correctional supervision than the control facilities (Pearson et al. 2014). Moreover, staff in the facilities that implemented the change team approach for improving the delivery of HIV services increased their perceptions of the value of HIV services as compared to staff in the control facilities (Visher et al. 2014). Staff in the facilities that participated in the change team activities rated implementing HIV services in their facility as more acceptable and feasible as compared with staff in the control facilities. Thus, the set of results from the current analysis indicating sustained changes in service outcomes implemented during the HIV-STIC study provides preliminary support for the use of a local change team approach to implementing evidence-based practices in criminal justice settings.

### Future directions

This study was conducted between six and nine months after the end of the implementation. However, there might be a delay between when changes have been sustained and when they can be observed. Moreover, the persistence of implemented changes may fluctuate over time. Future studies could look at the sustainability from a long-term perspective and assess which aspects of the implemented services are sustained, with a focus on how to maximize the investment and capacity of an implementation project.

It is important to distinguish the sustainability of implementation processes and strategies (e.g., interagency relationship, LCT model) from the sustainability of actual implementation outcomes. Even though they are two important pillars for a successful implementation, little attention has been paid to understand the extent to which implementation processes have been sustained and the pertinent factors that influence that sustainability. Future research could look at how implementation processes are sustained as well as how the organizational adoption of implementation processes serves as an instrument to enhancing the quality of health services.

## Abbreviations

CJ-DATS: Criminal Justice Drug Abuse Treatment Studies
HIV-STIC: HIV Services and Treatment Implementation in Corrections
LCT: Local Change Team
PI: Principal investigator
ES: Executive sponsor
